# A Sparsity-Promoted Decomposition for Compressed Fault Diagnosis of Roller Bearings

**DOI:** 10.3390/s16091524

**Published:** 2016-09-19

**Authors:** Huaqing Wang, Yanliang Ke, Liuyang Song, Gang Tang, Peng Chen

**Affiliations:** 1 College of Mechanical & Electrical Engineering, Beijing University of Chemical Technology Chao Yang District, Beijing 100029, China; hqwang@mail.buct.edu.cn (H.W.); 18649880645@163.com (Y.K.); 2 Graduate School of Bioresources, Mie University, 1577 Kurimamachiya-cho, Tsu, Mie 514-8507, Japan; chen@bio.mie-u.ac.jp

**Keywords:** roller bearing, fault diagnosis, compressed sensing, sparsity, fault feature, tunable Q-factor wavelet transform

## Abstract

The traditional approaches for condition monitoring of roller bearings are almost always achieved under Shannon sampling theorem conditions, leading to a big-data problem. The compressed sensing (CS) theory provides a new solution to the big-data problem. However, the vibration signals are insufficiently sparse and it is difficult to achieve sparsity using the conventional techniques, which impedes the application of CS theory. Therefore, it is of great significance to promote the sparsity when applying the CS theory to fault diagnosis of roller bearings. To increase the sparsity of vibration signals, a sparsity-promoted method called the tunable Q-factor wavelet transform based on decomposing the analyzed signals into transient impact components and high oscillation components is utilized in this work. The former become sparser than the raw signals with noise eliminated, whereas the latter include noise. Thus, the decomposed transient impact components replace the original signals for analysis. The CS theory is applied to extract the fault features without complete reconstruction, which means that the reconstruction can be completed when the components with interested frequencies are detected and the fault diagnosis can be achieved during the reconstruction procedure. The application cases prove that the CS theory assisted by the tunable Q-factor wavelet transform can successfully extract the fault features from the compressed samples.

## 1. Introduction

Since roller bearing are an integral component in rotating machinery, it is necessary to conduct condition monitoring for them, aiming at preventing the occurrence of unpredictable failures [1,2]. Vibration-based diagnostic techniques are the most effective and widely-used methods for state identification of roller bearings, as the vibration signals contain much dynamic information on machine status [2,3,4]. So far, the vibration-based diagnostic techniques can be broadly classified into three parts: time-domain analysis, frequency-domain analysis and time-frequency analysis. Various approaches corresponding to these types have been developed, such as time synchronous averaging, symptom parameters (time-domain analysis) [5,6,7], envelope analysis (frequency-domain analysis) [8], fractional Fourier transform, wavelet transform, empirical mode decomposition and short time Fourier transform (time-frequency analysis) [9,10,11,12,13,14,15,16,17].

The abovementioned approaches have received much attention. However, they are almost always achieved by sampling the vibration signals under the Shannon sampling theorem. Under this sampling strategy, much redundant information will be measured, which creates big-data when continuous condition monitoring is carried out. The big-data problem brings great pressure to data storage and transmission. Although current storage capability is adequate enough to record the large amount of vibration signals induced by the continuous condition monitoring under the Shannon sampling theorem, it does not work well in many cases. For example: (1) in some conditions, less time is required to make a quick evaluation of the running machinery from less transferred data. For example, for the online monitoring of flying aircraft engine, it would be better if less data were transferred between the flying aircraft and the ground supervision center and the running status can still be inferred from these compressed transferred data. This will take less time to process the compressed vibration signals and provide suggestions as quickly as possible both for the aircraft pilot and the monitoring system on ground; (2) in several cases, the economic costs of condition monitoring should be taken into account. The cost of the condition monitoring will be increased if a storage device with large storage capacity is used. If the big data can be compressed enough, less transmission devices and post-processing devices can be used, which can reduce the cost. Also, the big-data problem increases the difficulty of vibration-signal processing. Numerous investigations have been conducted on the data compression and several approaches have been developed, such as wavelet transform and cosine transform. The main idea of wavelet-transform-based data compression is to decompose the raw data at a predetermined level. Then a suitable threshold is selected to eliminate the interference and the data capacity can be compressed [18,19,20]. This data compression strategy has been widely used in imaging processing and power delivery, etc. However, the vibration signals of roller bearing cannot be presented in a sparse way in wavelet domain as well as cosine transform [21,22]. Therefore, it is difficult to determine a threshold to achieve the data compression. Thus, the studies related to the big-data problem have been attracting researchers in various fields.

In 2006, Candès et al. [23] proposed the CS theory and a milestone was reached in solving the big-data problem. In [23], they proved the mathematical principle that the raw signals can be reconstructed from under-sampled data. With a sparse representation and a well-designed measurement matrix, the original signals can be recovered from a very limited number of observations. Compared to the above-mentioned data compression approaches, the amount of the original signals can be reduced using the measurement matrix while the wavelet-transform-based data compression just to eliminate the useless features in the signals with the amount of signals unaltered. The down-sampled signals can be stored in the sending side and transmitted to receiving end. Moreover, the running time can be decreased with a less amount of signals when a fault diagnosis algorithm is utilized. Owing to the promising potential of CS theory, it has been widely used in numerous fields, such as image processing, remote sensing and medical field. Chan et al. [24] described a novel and high-speed pulsed terahertz Fourier imaging system based on CS theory. Wakin et al. [25] proposed a new hardware to support a new theory of compressed imaging by acquiring random projections of the signals. Kim et al. [26] applied the CS theory to magnetic resonance imaging. Ma [27] applied the CS theory to aerospace remote sensing aiming at reducing data acquisition and imaging cost.

Considering the multiple applications of CS theory, there is a high probability that the theory is applicable to the field of fault diagnosis. The fundamental idea of CS theory is that the vibration signals must be sparse enough to meet the requirement of sparsity. However, the vibration signals induced by the faulty bearings are inadequately sparse and it is difficult to select a suitable transformation to promote the sparsity of the vibration signals using the traditional transforms, such as Fourier transform and wavelet transform. Thus, the sparse representation becomes a major obstacle for the application of CS theory in roller bearing fault diagnosis.

To overcome the problem of inadequate sparsity, sparse representation has been receiving considerable attention. Sparse representation implies representing a signal as a linear combination of a few atoms of a dictionary. According to the applications, the dictionary can be divided into two categories: fixed dictionary and learning dictionary. The former, such as the dictionaries with Fourier basis or wavelet basis, can be used for various signals. However, the successful sparse representation cases are limited, which means it cannot decompose the signals sparsely. The latter is trained from some given atoms and the learning process is carried out according to the structure and characteristics of the target signals. Thus, the learning dictionary can decompose the signals in a sparse way. However, the trained dictionary are learned from the target signals, which makes it more suitable for a specific signal as it lacks adaptivity. Moreover, to establish a suitable dictionary for signal representation involves great computation, which makes it more difficult to detect the fault as soon as possible. 

Therefore, a sparsity-promoted method named the tunable Q-factor wavelet transform (TQWT) [28,29] is utilized in this work to increase the sparsity of vibration signals, which is beneficial to the application of CS theory. Then, a compressed fault detection strategy based on the TQWT and CS is proposed. Assisted by the TQWT, the original signals can be decomposed into two parts. One is the transient impact components, which contain the fault features with the sparsity promoted. Another is the high oscillatory components, which include the noise. Through the decomposition using the TQWT, the transient impact components replace the raw signals for analysis. In addition, the decomposed signals are sparser than the original signals, which promotes the application of CS theory. Furthermore, to our knowledge, the existence of noise in the vibration signals increases the difficulty of fault diagnosis of roller bearings as the significant fault features might be covered in heavy noise. To overcome this shortcoming, the noise in the original vibration signals can be eliminated by using the TQWT and the fault features can be enhanced, which can help to increase the sparsity of the target signals. With a given measurement matrix, the CS is utilized to extract fault features from low-dimensional data, which cannot meet the requirement of the Shannon sampling theory. With the help of the matching pursuit method, the components with the interested frequencies can be detected, according to which the operating condition of roller bearings can be evaluated. 

The rest of this paper is organized as follows: Section 2 introduces the theoretical background in this work, including the TQWT and the CS. Section 3 describes the compressed fault detection strategy. The application cases of the proposed method are presented in Section 4. The conclusions are drawn in Section 5.

## 2. Theoretical Background

### 2.1. Tunable Q-Factor Wavelet Transform 

The Q-factor can be acquired as follows, which is the ratio of its center frequency to its bandwidth [28,29,30,31,32,33]:

(1) $$Q=\frac{f_{o}}{BW}$$

**Figure 1:**
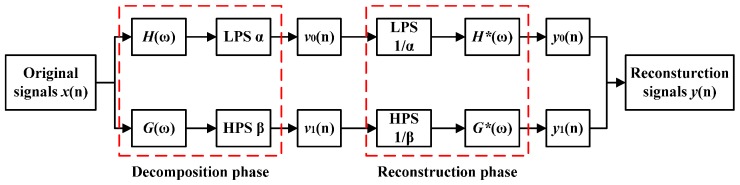
Two-channel filter bank.

Where *f*_0_ is the center frequency, and *BW* is the bandwidth of the analyzed signals, which can be determined by kurtogram of spectral kurtosis [34]. Q-factor reflects the oscillatory behavior of a signal. Generally, a high Q-factor is more suitable to process oscillatory signals, whereas a low Q-factor is usually utilized to process transient impact components. The traditional wavelet transform decomposes the signals with a constant Q-factor, which imposes a limitation on tuning the Q-factor to match different oscillatory behaviours of target signals. Furthermore, when using the traditional wavelet transform, the selection of the wavelet basis has a significant influence of the results. However, the TQWT is much more flexible, since the Q-factor can be easily turned for the concerned signals. The TQWT was first proposed by Selesnick [28] and it can be implemented using a sequence of two-channel filter bank as shown in Figure 1. 

In Figure 1, *x*(*n*) denotes the original signals. *H*(*ω*) and *G*(*ω*) represent the low-pass filter frequency response function and high-pass filter frequency response function, respectively. *α* and *β* stand for low-pass scaling parameter and high-pass scaling parameter, respectively. ***v***_0_(*n*) is the transient impact component, while ***v***_1_(*n*) is the high oscillation one [28,29,30,31,32,33]. 

For perfect reconstruction, the frequency response should be designed so that the reconstructed signal *y*(*n*) can be almost equal to *x*(*n*). Mathematically, the frequency response can be defined as follows:

(2) $$H(\omega )=\{\begin{array}{c} 1,\;\left|\omega \right|\leq (1-\beta )\pi .\\ \theta (\frac{\omega +(\beta -1)\pi }{\alpha +\beta -1}),\;(1-\beta )\pi \leq \left|\omega \right|<\alpha \pi \\ 0,\;\alpha \pi \leq \left|\omega \right|\leq \pi \end{array}\;$$

(3) $$G(\omega )=\{\begin{array}{c} 0,\;\left|\omega \right|\leq (1-\beta )\pi .\\ \theta (\frac{\alpha \pi -\omega }{\alpha +\beta -1}),\;(1-\beta )\pi \leq \left|\omega \right|<\alpha \pi \\ 1,\;\alpha \pi \leq \left|\omega \right|\leq \pi \end{array}$$

where: $0<\;\alpha <1,\;0<\beta \leq 1,\;\theta \left(\omega \right)=\frac{1}{2}\left(1+cos\omega \right)\sqrt{2-cos\omega }\text{ for}\;|\omega |\leq \pi .$

**Figure 2:**
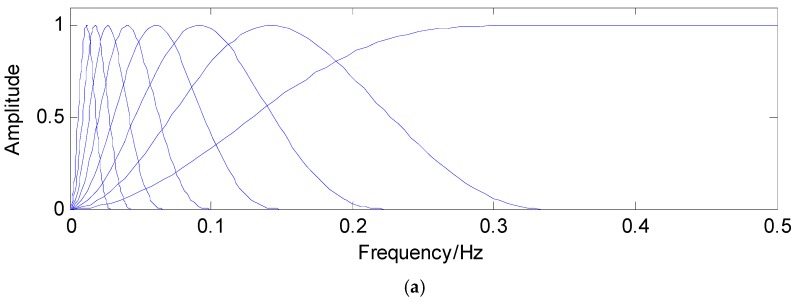

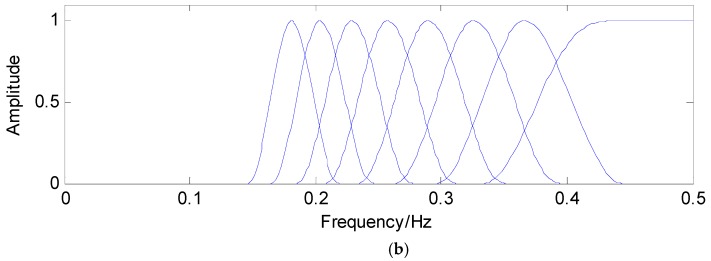
Frequency response with different Q-factor values (**a**) Q = 1, R = 3; (**b**) Q = 5, R = 3.

**Figure 3:**
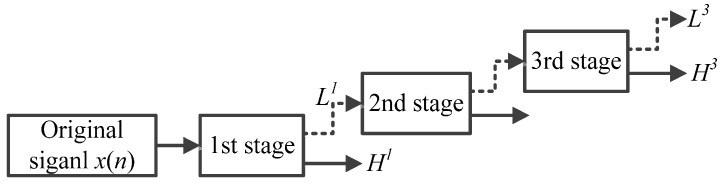
Wavelet transform with level *L* = 3.

Using different parameters, the TQWT can decompose the concerned signals into transient impact parts and high oscillatory parts. The main parameters in the TQWT are the Q-factor *Q*, the redundancy *r* and the stages (levels) *L*. According to the definition of Q-factor, it can be concluded that Q is a measure of the number of oscillations the wavelet exhibits. To our knowledge, the sustained oscillatory signals have narrow bandwidth and are corresponding to high Q-factor. On the contrary, the transient impact signals have low Q-factor. Frequency responses with different Q are shown in Figure 2. The parameter *r* denotes the redundancy of the TQWT through certain stages. The parameter *J* represents the number of filter banks. Figure 3 is an example of *L* = 3. When *L* is determined, *L* + 1 subbands can be obtained: the high-pass filter output of each filter bank and the low-pass filter output of the final filter bank [28,29,30,31,32,33].

When *Q* and *r* are determined, α and β can be calculated [28]:

(4) $$\{\begin{array}{c} \beta =\frac{2}{Q+1}\\ \alpha =1-\frac{\beta }{r} \end{array}\;$$

It is known that the vibration signals generated by faulty roller bearings always consist of the fault transient impact component and the oscillation component. Hence, the TQWT is appropriate for processing the vibration signals generated by fault bearings. 

Suppose that the original vibration signals measured by the sensors can be expressed as follows:

(5) $$x=x_{1}+x_{2}$$

where *x* is the original signals induced by the faulty bearings. *x*_1_ denotes the fault transient impact components, which conclude the fault features. *x*_2_ represents the high oscillation components, which are almost the surrounding noise.

Considering that TQWT_1_ and TQWT_2_ denote the TQWT with a high and a low value of Q-factor. Then the decomposition can be achieved by solving the following optimization problem:

(6) $$\{\begin{array}{c} \arg\;\underset{\omega_{1},\omega_{2}}{\min}\lambda_{1}{\left\|\omega_{1}\right\|}_{1}+\lambda_{2}{\left\|\omega_{2}\right\|}_{1}\\ x=TQW{T_{1}}^{-1}(\omega_{1})+TQWT_{2}^{-1}(\omega_{2}) \end{array}$$

To utilize the TQWT flexibly, the sub-band-dependent regularization will be used:

(7) $$\{\begin{array}{c} \arg\;\underset{\omega_{1},\omega_{2}}{\min}\sum_{j=1}^{J_{1}+1}\lambda_{1j}{\left\|\omega_{1j}\right\|}_{1}+\sum_{j=1}^{J_{2}+1}\lambda_{2j}{\left\|\omega_{2j}\right\|}_{1}\\ x=TQWT_{1}^{-1}(\omega_{1})+TQWT_{2}^{-1}(\omega_{2}) \end{array}$$

where $\omega_{ij}$ represents the sub-band *j* of TQWT*_i_*. When $\omega_{1}$ and $\omega_{2}$ are determined, the *x*_1_ and *x*_2_ can be gained as follows:

(8) $$\{\begin{array}{c} x_{1}=TQWT_{1}^{-1}(\omega_{1})\\ x_{2}=TQWT_{2}^{-1}(\omega_{2}) \end{array}$$

It is well known that the vibration signals measured from the faulty rotating machinery are usually flooded by the noise. Therefore, the representation of the original signals can be modified as follows:

(9) $$y=x_{1}+x_{2}+n$$

Then, the solution can be changed into the following model:

(10) $$\arg\;\underset{\omega_{1},\omega_{2}}{\min}\left\|y-\Phi_{1}\omega_{1}-\Phi_{2}\omega_{2}\right\|+\sum_{j=1}^{J_{1}+1}\lambda_{1j}{\left\|\omega_{1j}\right\|}_{1}+\sum_{j=1}^{J_{2}+1}\lambda_{2j}{\left\|\omega_{2j}\right\|}_{1}$$

where $\Phi_{1}$ represents the inverse TQWT with a high value of Q-factor, while $\Phi_{2}$ denotes the inverse TQWT with a low value of Q-factor.

### 2.2. Basic Idea of the Compressed Sensing Theory

In the fault diagnosis of roller bearings, data-collection under the Shannon sampling theorem results in the big-data problem and the large amount of vibration signals increases the cost of data storage and puts great pressure on signal processing. Furthermore, the fault diagnosis needs timely detection and the big data increase the time of fault diagnosis, which means there is a contradiction between the detection accuracy and efficiency.

To overcome the problems induced by the big data, the CS developed, which can ease the pressure of signal processing by reconstructing a signal from limited samples [35,36,37,38,39,40,41,42].

Provided $x_{1}\left(t\right)$ is a N×1 transient impact signals obtained by the TQWT with a low Q-factor, which can decompose the original signals in a sparse way. In other words, $x_{1}\left(t\right)$ can be expressed by a group of N×1 basis [36,37]:

(11) $$x_{1}(t)=\Psi \alpha$$

where $\Psi =\left\{\psi_{1},\psi_{2},\cdots ,\psi_{N}\right\}\in N\times N$ is the basis vector. The representation coefficients are denoted by α.

The signals can be considered *K*-sparse, when the representation coefficients α contain only *K* non-zeros (K≪N). Then, a measurement matrix works as a compressor to reduce the dimension of the target signals [43,44]:

(12) $$y=\Phi x_{1}(t)$$

so:

(13) $$y=\Phi \Psi \alpha =A^{CS}\alpha$$

where Φ is a M×N(M≪N) measurement matrix. $A^{cs}$ is called the sensing matrix. The N×1 signal can be compressed to a M×1 signal using a measurement matrix according to Equation (12).

The accurate sparse solution may be pursued by a $\ell_{0}-norm$ problem:

(14) $$\min{||\alpha ||}_{0}\;s.t.\;y=A^{CS}\alpha$$

However, the above solution is a *NP*-hard problem. Thus, the solution is achieved by $\ell_{1}-norm$ optimization:

(15) $$\min{||\alpha ||}_{1}\;s.t.\;y=A^{CS}\alpha$$

When $A^{cs}$ meets the principle of restricted isometry property (RIP) as follows, the raw signals can be reconstructed with small error:

(16) $$\left(1-\varepsilon \right){\left\|x_{1}\right\|}_{2}^{2}\leq {\left\|\Phi \Psi x_{1}\right\|}_{2}^{2}\leq \left(1+\varepsilon \right){\left\|x_{1}\right\|}_{2}^{2}$$

where ε∈(0,1) and its equivalent condition is Φ should be uncorrelated with Ψ.

## 3. The Proposed Fault Detection Method

**Figure 4:**
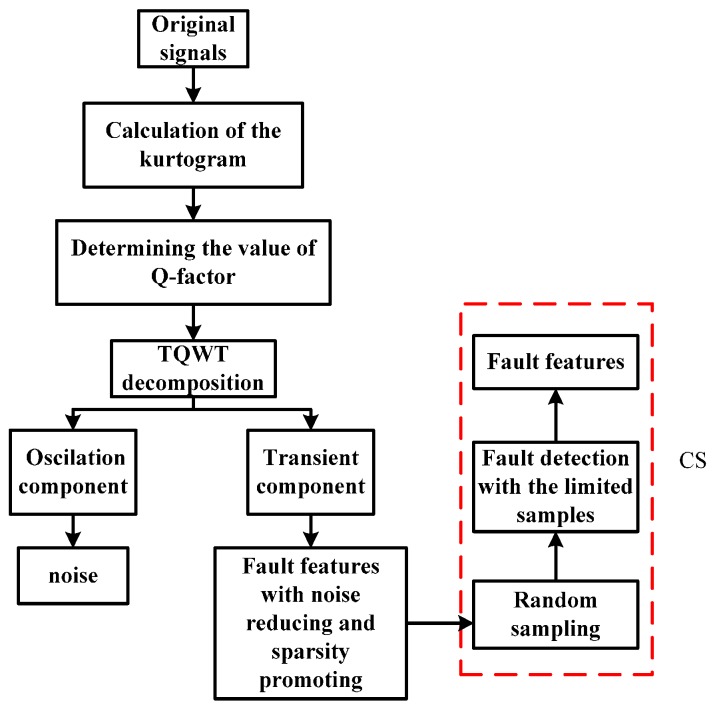
The proposed fault detection method based on TQWT and CS.

**Figure 5:**
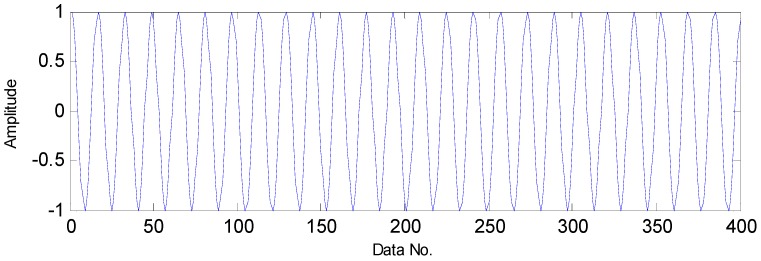
The simulated harmonic signal.

**Figure 6:**
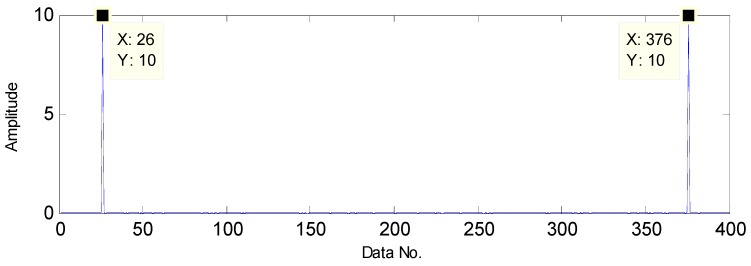
The sparsity of the harmonic signal in the Fourier domain.

The flow diagram of the proposed method is presented in Figure 4. First, the polluted vibration signals measured from a faulty bearing are decomposed by the TQWT, through which high oscillation components and transient impact components can be obtained with the former containing the noise and the latter containing fault features. Moreover, the TQWT can decompose the raw signals in a sparse way and the decomposed signals are sparser than before as a result of the noise reduction, which is suitable for the application of CS theory. Then, the transient components are used to replace the original signals in the compressed-sensing-based signal processing in order to extract the fault features. It is known that when a failure occurs in an operating bearing, a certain frequency named the fault characteristic frequency generates, which can be decomposed into a series of harmonic signals. To investigate the sparsity performance of the harmonic signal in Fourier domain, a simulated signal is generated with a frequency of 50 Hz. The simulated signal and its sparsity are presented in Figure 5 and Figure 6, respectively. Thus, a fault detection strategy can be formulated that a harmonic signal with interested frequency can be detected instead of directly detecting the existence of the fault characteristic frequency.

Before performing TQWT, two Q-factor values should be determined. Since the kurtogram can represent the bandwidth and the center frequency of a signal, it is employed to calculate the high Q-factor. The low Q-factor is artificially set to be 1.5 by experience. When performing the TQWT to the original signals, the decomposed transient impact signals will be sparser with noise reduced, which makes it easier to detect the harmonic signal of interest. To meet the requirement of RIP principle, Gaussian random matrix is selected as measurement matrix for dimension reduction, which has low correlation with the Fourier basis [39,42,45]. The elements in Gaussian random matrix Φ∈M×N (M≪N) obey the Gaussian distribution with a mean of zero and a variance of 1/M. Each row of the Gaussian random matrix works like a sensor to measure the original vibration signals randomly. Assisted by the Gaussian random matrix, the concerned signals can be projected form an original dimension N to a lower dimension M. With a sparse representation and a well-designed measurement matrix, the faults can be detected with a high possibility. Finally, the harmonic signals with the interested frequency can be detected with limited samples far below the Nyquist sampling rate.

## 4. Application Cases

**Figure 7:**
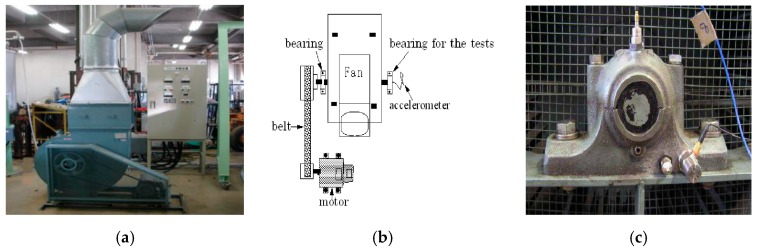
(**a**) Fan system (**b**) Flow diagram of the fan system (**c**) Location of the sensors.

**Figure 8:**
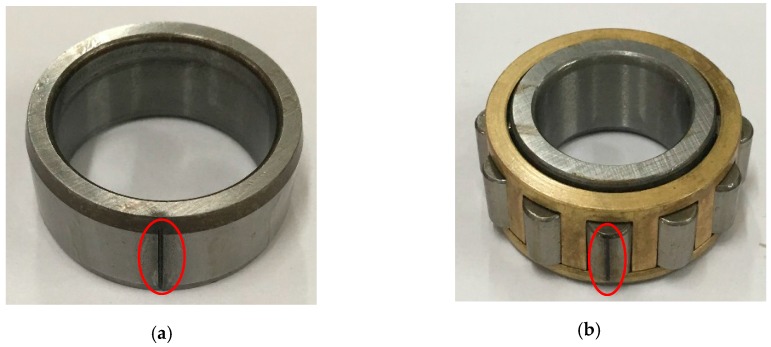
(**a**) Inner-race fault (**b**) rolling-element fault.

The vibration signals of roller bearing faults are measured through accelerometers. The faulty bearings are placed in a fan system which consists of a motor, a belt, a fan and a couple of bearings, as shown in Figure 7a. The flow diagram of the fan system is presented in Figure 7b. The sensors are installed on the bearing housing as shown in Figure 7c, where the collected vibration signals might be valid and easily measured. The faulty roller bearings are presented in Figure 8a,b. In all experiments, the sampling frequency is 100 KHz and the roller bearings are at a speed of 500 rpm. The fault characteristic frequencies can be computed according to Equations (17)–(19) and the results are shown in Table 1 [46,47].

The fault characteristic frequency of the outer race:

(17) $$f_{o}=\frac{z}{2}(1-\frac{d}{D}\cos\alpha )f_{r}$$

The fault characteristic frequency of the inner race:

(18) $$f_{i}=\frac{z}{2}(1+\frac{d}{D}\cos\alpha )f_{r}$$

The fault characteristic frequency of the rolling element:

(19) $$f_{b}=\frac{D}{2d}(1-(\frac{d}{D}\cos\alpha {)}^{2})f_{r}$$

where *Z* denotes the number of roller elements, *f*_r_ is the rotating frequency, *d* is the roller diameter, *D* represents the pitch diameter and α is the contact angle.

### 4.1. Detection of the Bearing with an Inner-Race Fault

**Figure 9:**
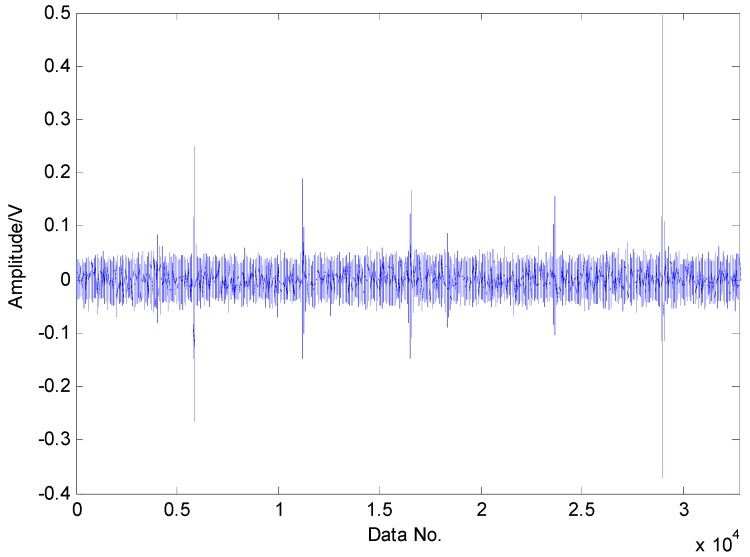
Original vibration signals collected from roller bearing with an inner-race fault.

**Figure 10:**
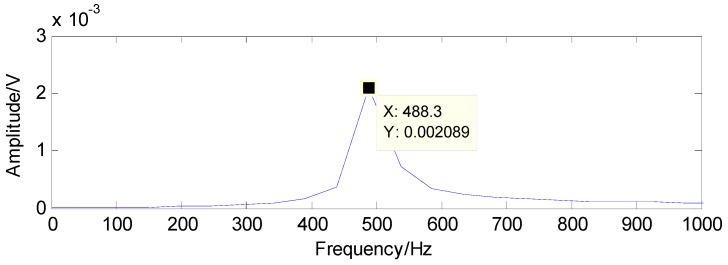
First detection result using the raw signals based on the method in [37].

**Figure 11:**
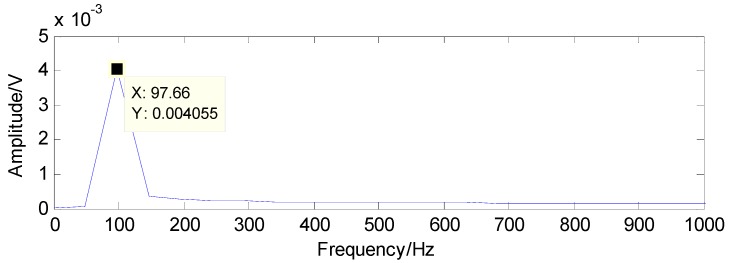
Second detection result using the raw signals based on the method in [37].

The proposed method is first utilized to extract the fault features of roller bearing with a single fault in the inner race. The experimental results are presented as follows: as presented in Figure 9, the original vibration signals contain noise and the amount of vibration signals for fault diagnosis is a bit larger, which increases the difficulty of fault diagnosis. Moreover, the original vibration signals are inadequately sparse, which is a major obstacle for the application of CS. Applying CS to the original signals using the method in reference [37], we cannot extract the fault features successfully in the first two attempts due to the inadequate sparsity as well as the interference, as shown in Figure 10 and Figure 11. The fault features might be detected through a few more attempts, however, it wastes lots of time in this way. Therefore, the TQWT is regarded as a sparsity-promoted approach to represent the analyzed signal in a sparse way and to reduce the influence of noise as well, which can make it much easier to detect the fault features.

**Figure 12:**
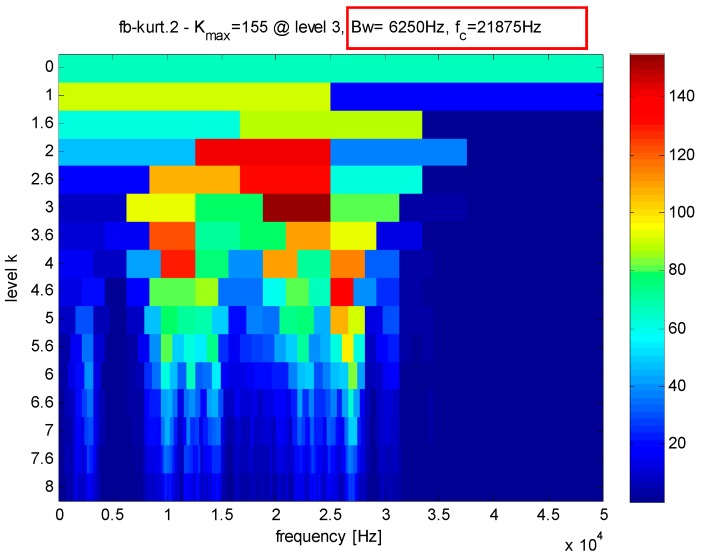
Kurtogram of the signals as shown in Figure 7.

**Figure 13:**
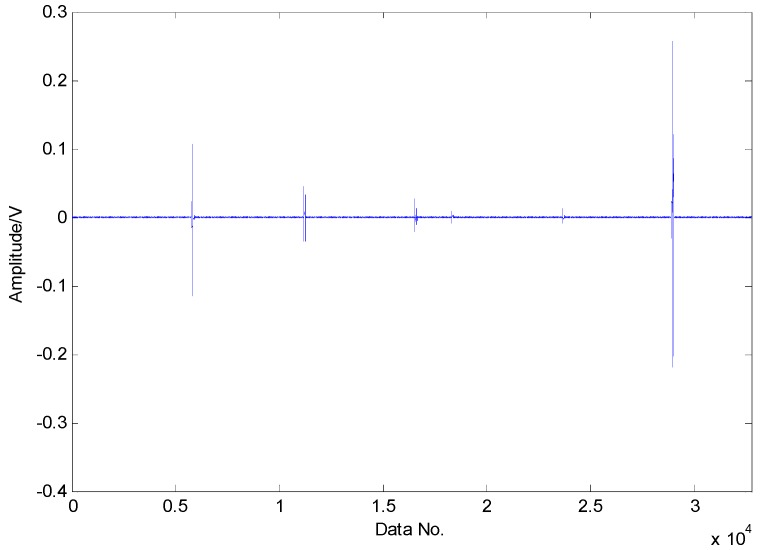
The transient impact component using TQWT.

**Figure 14:**
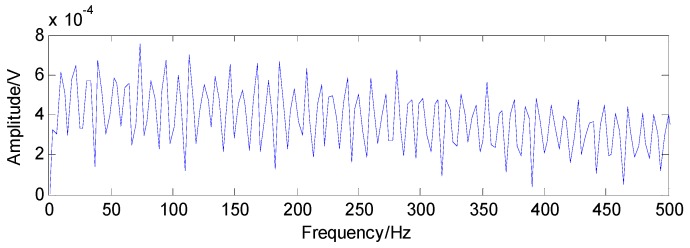
Envelope spectrum of the transient impact component in Figure 11.

From Figure 12, the bandwidth and the center frequency of analyzed signals can be determined as 6250 Hz and 21,875 Hz, respectively. According to Equation (1), the value of the Q-factor can be obtained as 3.5 and the other Q-factor is artificially set to 1.5 by experience. The transient component containing the fault features can be gained through the TQWT by discarding the high oscillation component, as shown in Figure 13. Compared with the original signals, the noise is reduced and the decomposed signals are much sparser, which is beneficial to the application of the CS. The envelope spectrum of the transient impact component in Figure 14 indicates that the fault features still cannot be extracted using the envelope analysis.

**Figure 15:**
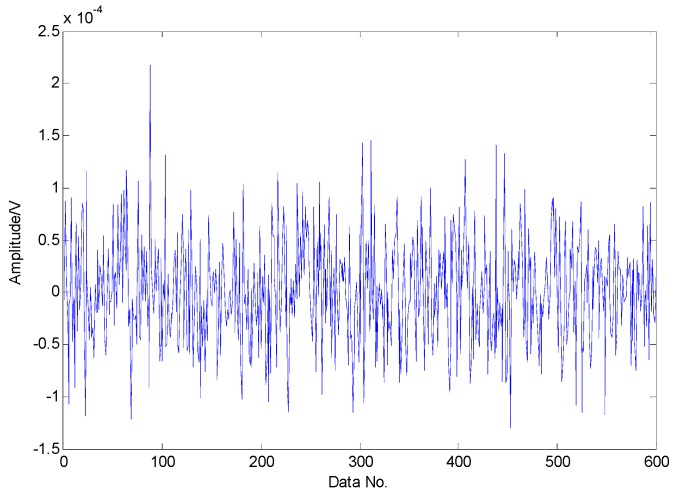
Random sampling of transient impact component through Gaussian random matrix.

**Figure 16:**
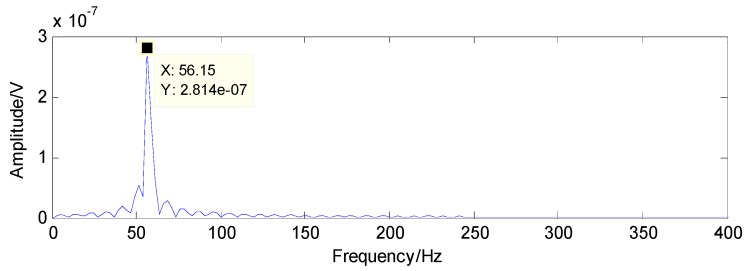
The detected fault characteristic frequency through matching pursuit.

**Figure 17:**
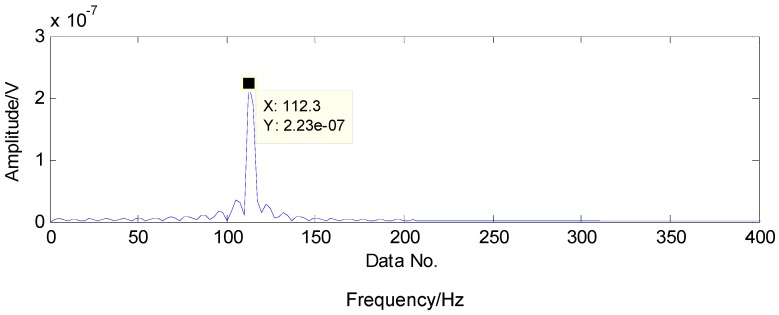
Second harmonic of the detected fault characteristic frequency through matching pursuit.

To extract the fault features, the CS theory is employed to detect the failure from limited sampling points and the amount of the analysed signals is compressed to 600 sampling points as shown in Figure 15 through random sampling assisted by a Gaussian random matrix. It is well-known that once there is a local defect in the roller bearing, a force impulse occurs when the surface with fault strikes to another surface, leading to a resonance phenomenon. The resonance frequency is about several thousand Hertz. To meet Shannon sampling theorem, the sampling frequency should be big enough. That is to say, the amount of sampling points for signal processing should be much more than 600. However, the amount of target signals used in the proposed method are much less than that of traditional methods based on Shannon sampling theorem and the fault features can be extracted as predicted by the matching pursuit in the proposed method. As presented in Table 1, the theoretical fault characteristic frequency of inner-race fault is 56.09 Hz and the twice value is 112.18 Hz. The detected results through TQWT-based CS theory are 56.15 Hz and 112.3 Hz, as presented in Figure 16 and Figure 17, respectively. Thus, since the detected results are almost equal to the theoretical ones, a conclusion can be drawn that there is a fault in the inner race of this roller bearing.

### 4.2. Detection of the Bearing with an Rolling Element Fault

**Figure 18:**
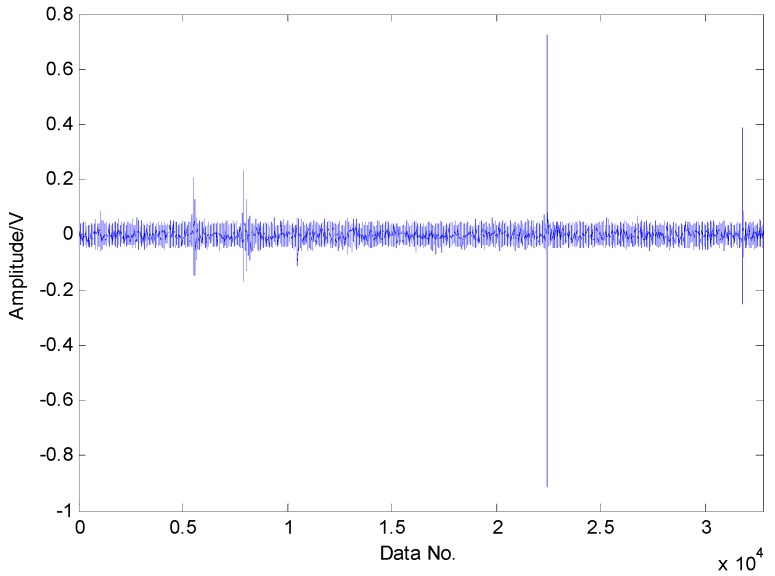
Original vibration signals collected from roller bearing with a rolling-element fault.

To fully validate the effectiveness of the proposed fault detection strategy, a roller bearing with a faulty rolling element was employed. From the experimental results in Figure 18, a conclusion can be drawn that the noise in the raw vibration signals is so strong that the fault features are submerged by the heavy noise.

**Figure 19:**
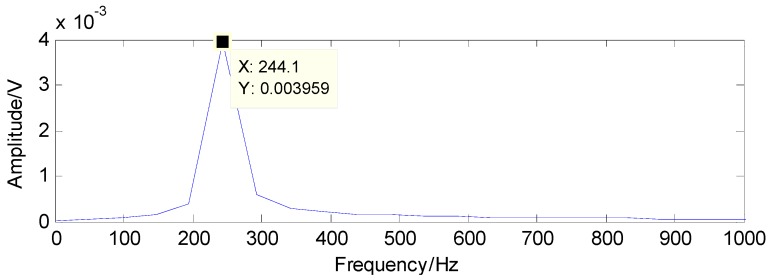
First detection result using the raw signals based on the method in [37].

**Figure 20:**
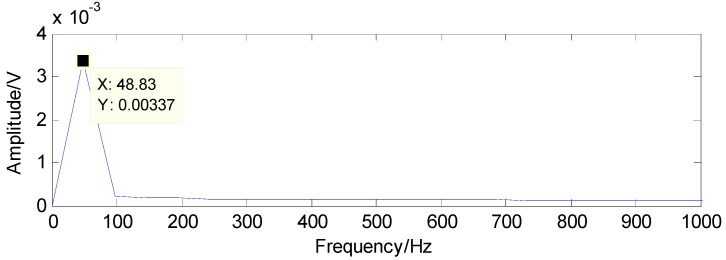
Second detection result using the raw signals based on the method in [37].

The results detected through the method in [37] are presented in Figure 19 and Figure 20. Compared with the theoretical values of rolling-element fault features, the first detected result in Figure 19 and the second detected result in Figure 20 are not the fault features of rolling-element fault, according to which the existence of rolling-element fault cannot be determined

**Figure 21:**
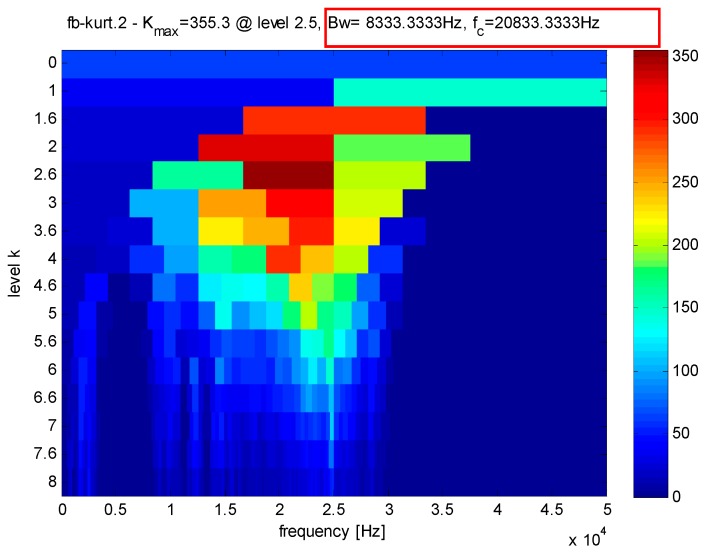
Kurtogram of the signals as shown in Figure 16.

**Figure 22:**
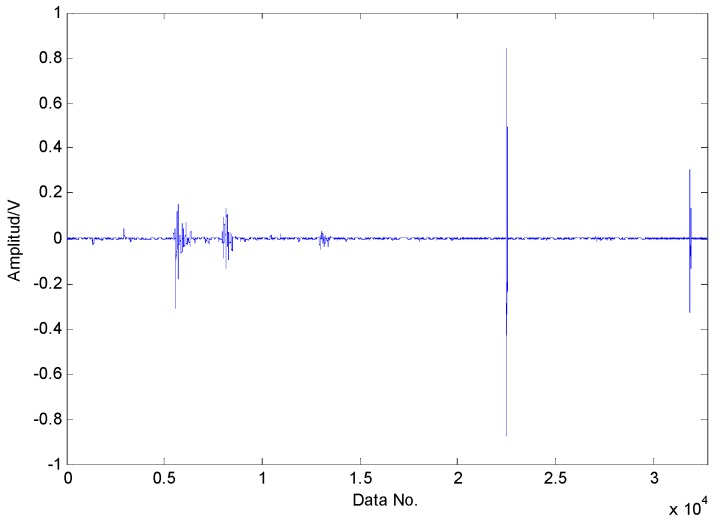
The transient impact component using TQWT.

**Figure 23:**
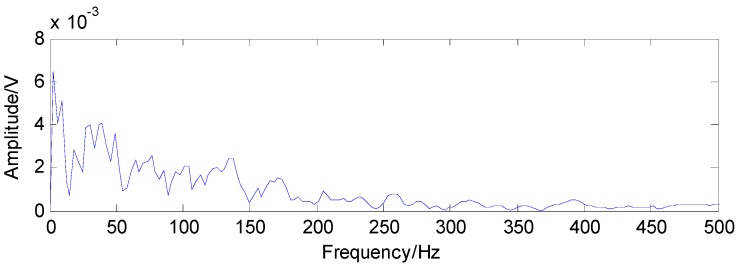
Envelope spectrum of the transient impact component in Figure 20.

Assisted by the kurtogram of the vibration signals in Figure 21, the high Q-factor can be determined as 2.5 and the low Q-factor is artificially determined as 1.5 by experience. As presented in Figure 22, the decomposed transient impact components are much sparser than before and the noise are mostly eliminated. To highlight advantage of the proposed method, the envelope analysis is utilized as a comparison. However, the fault features cannot be extracted through envelope spectrum in Figure 23. Thus, the proposed TQWT-based CS method is utilized to perform the fault detection.

**Figure 24:**
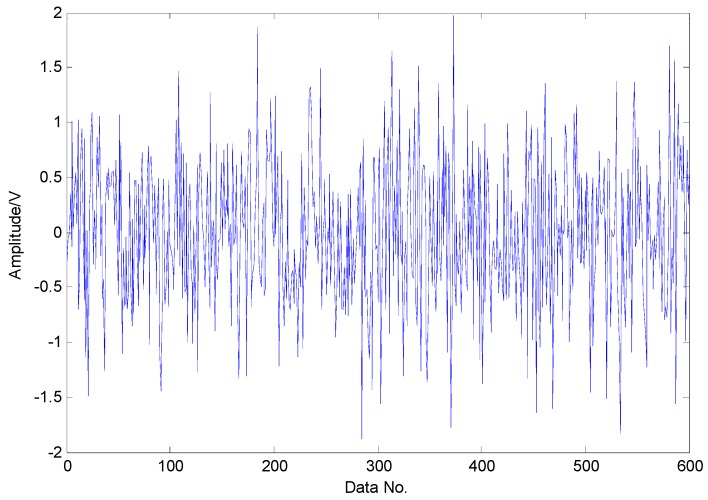
Random sampling of transient impact component through Gaussian random matrix.

A Gaussian random matrix is used as measurement matrix to acquire low-dimension signals, as shown in Figure 24. 

**Figure 25:**
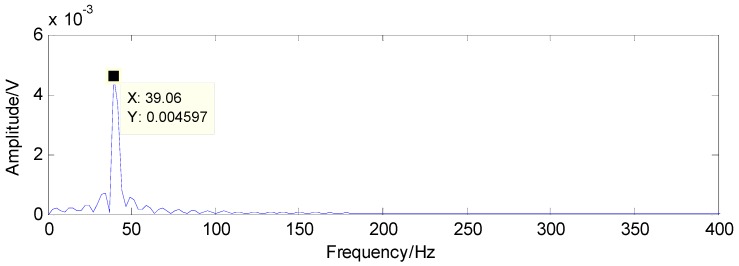
The detected fault characteristic frequency through matching pursuit.

**Figure 26:**
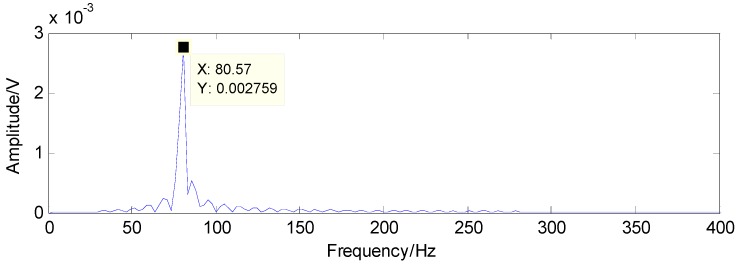
Second harmonic of the detected fault characteristic frequency through matching pursuit.

To successfully extract the fault features, the matching pursuit is utilized and the detected results can be acquired through the matching pursuit, as presented in Figure 25 and Figure 26, containing the fault characteristic frequency and its twice value, which are almost equal to the theoretical values in Table 1. Thus, the judgment that there is a rolling-element fault in this roller bearing can be concluded.

### 4.3. Detection of the Healthy Bearing

**Figure 27:**
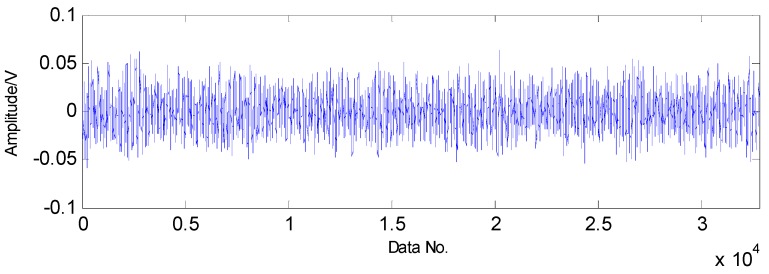
Original vibration signals collected from healthy roller bearing.

**Figure 28:**
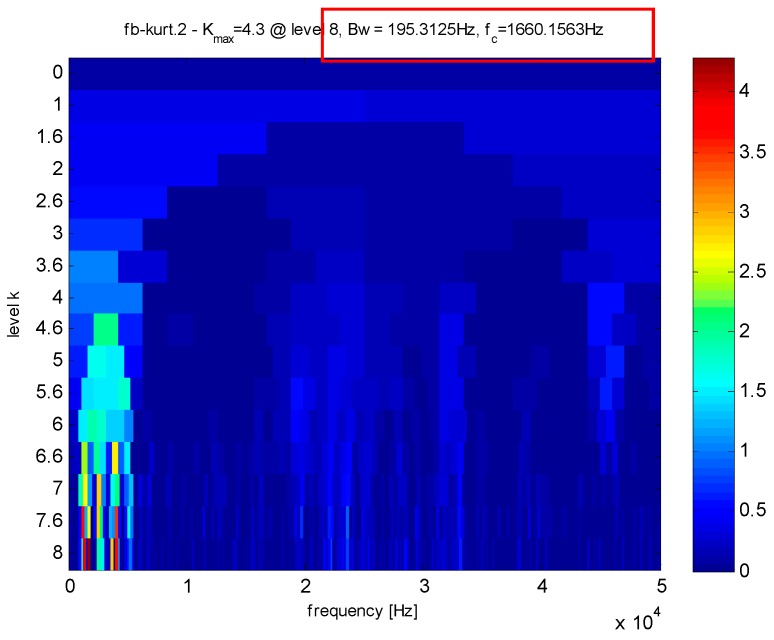
Kurtogram of the signals as shown in Figure 25.

**Figure 29:**
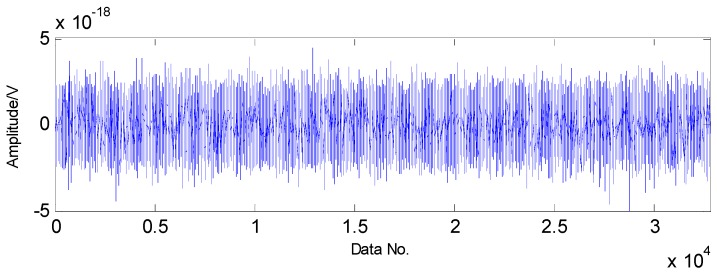
The transient impact component using TQWT.

**Figure 30:**
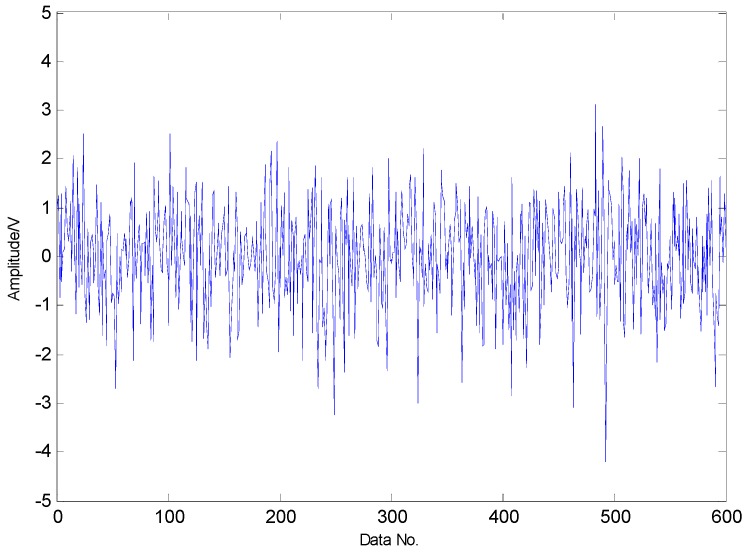
Random sampling of transient impact component through Gaussian random matrix.

**Figure 31:**
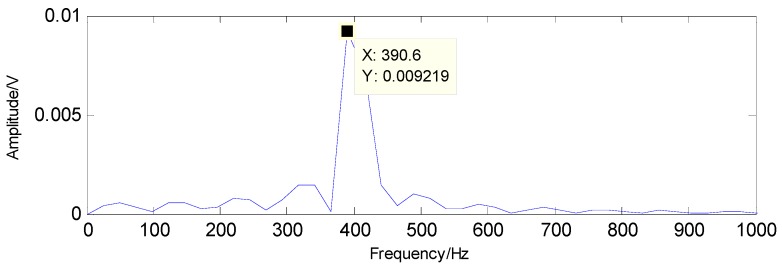
The detected fault characteristic frequency through matching pursuit.

**Figure 32:**
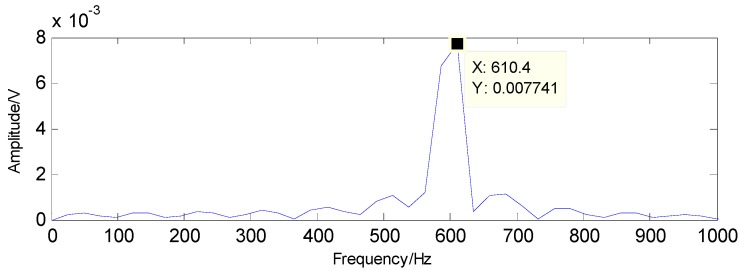
Second harmonic of the detected fault characteristic frequency through matching pursuit.

In this part, the vibration signals from healthy roller bearing are utilized to show that the proposed method does not suffer from false alarms. The collected healthy data are presented in Figure 27 and the kurtogram in Figure 28 shows that the Q-factor can be determined as 8.5, which is the ratio of 1660.1583 Hz to 195.3125 Hz. Another Q-factor is also set to 1.5. Through the TQWT, the transient impact component can be obtained as shown in Figure 29 and the sampling points of the vibration signals can be compressed to 600 as presented in Figure 30. Using matching pursuit, the frequencies can be detected as shown in Figure 31 and Figure 32. However, they are not the fault characteristic frequency and its second harmonic, which means that no fault exists. Thus, a conclusion can be drawn that this roller bearing is in normal status. 

## 5. Conclusions

A TQWT-based CS method is utilized in this work to extract the fault features of roller bearings. To overcome the inadequate sparsity when the CS is performed, TQWT is employed to promote the sparsity of vibration signals while reducing the interference. Kurtograms are employed to determine the Q-factor. Through TQWT, the original vibration signals can be decomposed into transient impact component and high oscillation component. The former contains fault features and the latter is almost full of noise. Thus, the transient impact component can be used to represent the raw signals for diagnosis and the high oscillation component is eliminated. In the application of CS, Gaussian random matrix is selected as the measurement matrix to obtain compressed signals and the matching pursuit is employed to detect the fault features, based on which the performance of roller bearing can be determined. Furthermore, the roller bearing faults at 800 rpm and 1300 rpm have also utilized to validate the effectiveness of the proposed method and the experimental results show that the proposed method can successfully assess the performance of roller bearing. 

## Figures and Tables

**Table 1:** Fault characteristic frequency.

Fault Location	Inner-Race	Rolling Element
Fault characteristic frequency (Hz)	56.09	39.33
Twice value (Hz)	112.18	78.66
